# Depletion of AADC activity in caudate nucleus and putamen of Parkinson’s disease patients; implications for ongoing AAV2-AADC gene therapy trial

**DOI:** 10.1371/journal.pone.0169965

**Published:** 2017-02-06

**Authors:** Agnieszka Ciesielska, Lluis Samaranch, Waldy San Sebastian, Dennis W. Dickson, Samuel Goldman, John Forsayeth, Krystof S. Bankiewicz

**Affiliations:** 1 Department of Neurological Surgery, University of California San Francisco, San Francisco, CA, United States of America; 2 Mayo Clinic, Jacksonville, FL, United States of America; 3 Department of Neurology, University of California San Francisco, San Francisco, CA, United States of America; University of South Florida, UNITED STATES

## Abstract

In Parkinson’s disease (PD), aromatic L-amino acid decarboxylase (AADC) is the rate-limiting enzyme in the conversion of L-DOPA (Sinemet) to dopamine (DA). Previous studies in PD animal models demonstrated that lesion of dopaminergic neurons is associated with profound loss of AADC activity in the striatum, blocking efficient conversion of L-DOPA to DA. Relatively few studies have directly analyzed AADC in PD brains. Thus, the aim of this study was to gain a better understanding of regional changes in AADC activity, DA, serotonin and their monoamine metabolites in the striatum of PD patients and experimentally lesioned animals (rat and MPTP-treated nonhuman primate, NHP). Striatal AADC activity was determined *post mortem* in neuropathologically confirmed PD subjects, animal models and controls. A regional analysis was performed for striatal AADC activity and monoamine levels in NHP tissue. Interestingly, analysis of putaminal AADC activity revealed that control human striatum contained much less AADC activity than rat and NHP striata. Moreover, a dramatic loss of AADC activity in PD striatum compared to controls was detected. In MPTP-treated NHP, caudate nucleus was almost as greatly affected as putamen, although mean DA turnover was higher in caudate nucleus. Similarly, DA and DA metabolites were dramatically reduced in different regions of PD brains, including caudate nucleus, whereas serotonin was relatively spared. After L-DOPA administration in MPTP-treated NHP, very poor conversion to DA was detected, suggesting that AADC in NHP nigrostriatal fibers is mainly responsible for L-DOPA to DA conversion. These data support further the rationale behind viral gene therapy with AAV2-hAADC to restore AADC levels in putamen and suggest further the advisability of expanding vector delivery to include coverage of anterior putamen and the caudate nucleus.

## Introduction

Progressive degeneration of dopaminergic innervation of the striatum underlies many of the motor and affective symptoms of Parkinson’s disease (PD). This decline is associated with fading efficacy of various anti-PD pharmaceuticals [1, 2], of which the most impactful is blunting of response to the drug L-DOPA, the immediate biosynthetic precursor of dopamine (DA). Initially, patients respond well to L-DOPA or Sinemet (L-DOPA/carbidopa). However, steadily increasing doses are required to maintain efficacy until the drug fails to elicit significant clinical response [3] and is earlier associated with motor fluctuations as well as L-DOPA-induced dyskinesia [4]. Previously, we have shown that animal models of PD respond poorly to L-DOPA and *post-mortem* striatal tissues have very low levels of DA even in the face of high L-DOPA concentrations [5–7]. Thus, weak behavioral response to drug appears to be driven by inefficient conversion of L-DOPA to DA [7]. This inefficiency can be explained by the substantial loss of the endogenous rate-limiting enzyme, aromatic L-amino acid decarboxylase (AADC), responsible for catalyzing decarboxylation of L-DOPA to generate DA. We found that, after infusion into parkinsonian nonhuman primate (NHP) putamen, an adeno-associated viral vector carrying human AADC gene (AAV2-hAADC) directed strong expression of AADC in medium spiny neurons and caused a 10- to 20-fold improvement in behavioral response to L-DOPA [8, 9]. This approach was translated into a clinical study in PD patients with encouraging results (NCT00229736) [10–12]. In that study, the post-commissural putamen was stereotactically targeted because DA depletion in this region is associated with motor disability in PD and imaging studies suggested a more pronounced disease progression in putamen than in caudate nucleus [13]. However, there is a paucity of information regarding monoamine content and AADC activity in the PD striatum. Therefore, in this study we measured striatal AADC activity and monoamine (DA, serotonin and their metabolites) concentrations in *post-mortem* samples of age-matched control and PD subjects as well as in experimental models that mimic PD. Previous studies report that striatal serotonin levels are relatively spared in PD [14]. Thus, we analyzed serotonin levels that served as a control for *post-mortem* human tissue degradation.

We show here that both caudate nucleus and putamen of PD patients are almost equally depleted of AADC activity relative to age-matched controls. A similar depletion was found in the striatum of 6-hydroxydopamine (6-OHDA)-lesioned rats and 1-methyl-4-phenyl-1,2,3,6-tetrahydropyridine (MPTP)-lesioned NHP. These data suggest that the strategy of solely targeting post-commissural putamen for gene therapy with AAV2-hAADC may be unnecessarily limited and that further clinical study should include vector infusions into caudate nucleus, especially considering the non-motor aspects of the disease.

## Material and methods

### Experimental animal models

All procedures were carried out with the approval of the Institutional Animal Care and Use Committee (IACUC), and in accordance with the Standard Operating Procedures protocol at the University of California San Francisco.

#### 6-OHDA rodent model

Ten male Sprague-Dawley rats (250–300 g, Charles River Laboratory) were caged with free access to food and water in groups of 3 with 12:12 h light/dark cycle. Briefly, animals received a stereotactically-guided injection of 6-OHDA into the medial forebrain bundle (MFB) in the right hemisphere as previously described [15]. Four weeks after 6-OHDA intoxication, full body rotations were recorded over 60-min periods and the data expressed as net full body turns/min, as previously described [15]. Seven rats exhibiting a rate >5 turns/min in response to 0.05-mg/kg subcutaneous apomorphine (www.sigmaaldrich.com) were included in the study. Animals were euthanized 2 days after rotational tests by transcardial perfusion of PBS and striata. Striata were dissected, weighed and stored at -80°C until processed for subsequent biochemical analyses. A group of naïve animals was included as a control group (n = 7).

#### MPTP nonhuman primate model

A total of 9 male and female NHP (Rhesus macaques; 8–14 kg; 6–12 years), were included in this study under a protocol approved by the University of California San Francisco IACUC. Seven out of 9 animals were lesioned with MPTP as previously described [7, 8, 16] and 2 naïve animals served as controls. Parkinsonism was induced by the administration of a single unilateral intracarotid artery (ICA) injection of MPTP (2–3 mg) combined with repeated intravenous injections of MPTP until the necessary degree of parkinsonism was achieved. Then, animals were evaluated with a behavioral rating scale (CRS) [7, 17] weekly until they began to show bilateral signs of parkinsonism (CRS>20). Animals developed a nearly complete dopaminergic lesion on the side of the ICA–comparable to an advanced stage of PD–and a partial lesion in the contralateral hemisphere–comparable to an earlier stage of PD. Once the primate parkinsonian model was established and the lesion stable, animals were randomly assigned to experimental groups. Two MPTP-treated animals were used to analyze AADC activity and monoamine levels and compared to the control group. Three MPTP-treated animals were used to analyze monoamine levels after receiving 3 different single oral doses of Sinemet prescription (100 mg L-DOPA/10 mg carbidopa (n = 1), 250 mg L-DOPA/25 mg carbidopa (n = 1) and 350 mg L-DOPA/35 mg carbidopa (n = 1), respectively), and compared with a MPTP-treated animal receiving no Sinemet treatment (n = 1) and with a MPTP-treated animal receiving chronically (200 mg L-DOPA/50 mg carbidopa daily for 2 months (n = 1)).

Survival time ranged between 6 and 14 months after the last intravenous injection of MPTP and those animals that were treated with Sinemet were necropsied 45 min after the last oral administration of L-DOPA.

Animals were euthanized by an intravenous overdose of sodium pentobarbital solution (>86 mg/kg) and then transcardially perfused with cold PBS. Brains were harvested, and coronally blocked into 3-mm blocks. Then, alternating blocks were snap-frozen in dry ice cooled isopentane and stored at -80°C until processing for biochemical analyses.

### Human brain tissue

*Post-mortem* brain tissue was obtained from 12 neuropathologically confirmed cases of PD and 10 age-matched control subjects without evidence of neurological abnormalities. De-identified samples were provided by the tissue bank at The Parkinson’s Institute (Sunnyvale, CA). No statistically significant differences on the age of death and *post-mortem* interval (time between death and freezing of brain tissue) were found between controls and PD samples (p>0.05; Table 1).

**Table 1:** Parkinson’s disease patients and control subject data.

PD Patients				Control Subjects			
	Gender	Age	PMI (h)		Gender	Age	PMI (h)
1	F	68	11.5	1	F	70	7
2	F	84	7.5	2	F	72	9.5
3	F	84	9	3	F	87	8.5
4	M	72	6.5	4	F	78	14
5	M	76	15	5	F	73	11
6	M	81	12.5	6	M	86	8
7	M	82	10.5	7	M	98	8
8	M	84	7.5	8	M	70	9.5
**Mean ± SD**		78.9 ± 6.2	10.0 ± 2.9	**Mean ± SD**		79.2 ± 10.1	9.4 ± 2.2

PMI: Post-mortem interval, expressed in hours (h); F: female; M: male; PD: Parkinson’s disease; SD: standard deviation.

### Biochemical assays

#### Frozen tissue sampling

Entire fresh frozen striata were used for biochemical analyses in rats, whereas brain punches were taken from specific regions of frozen NHP and frozen coronal human brain blocks. In L-DOPA-treated parkinsonian NHP, samples were collected from dorsal and ventral caudate (dC and vC, respectively), dorsal and ventral putamen (dP and vP, respectively), and nucleus accumbens (acM). Occipital cortex (oC) and temporal cortex (tC) were included as controls. Only dC and vC samples were collected from parkinsonian NHP and control that did not receive L-DOPA. Human brain punches were taken from anterior caudate (aC), anterior dorsal putamen (adP), anterior ventral putamen (avP), posterior dorsal putamen (pdP) and posterior ventral putamen (pvP) and oC, as a control.

#### AADC activity and monoamines levels

AADC activity was measured in samples from hemi-parkinsonian rats, NHP without L-DOPA treatment and humans. Enzymatic conversion of L-DOPA into DA in homogenates was performed as previously described [15, 18, 19]. Briefly, samples were homogenized by mechanical disruption in 0.32M sucrose (1:5 *w/w* of tissue). Samples were incubated at 37°C for 5 min and then, 0.3 mM L-DOPA was added to each sample. After 40 min, ice-cold 0.4 M perchloric acid (PCA) was added, the mixture centrifuged, and the enzymatic conversion of L-DOPA into DA was analyzed in the supernatant by HPLC.

Monoamines and their metabolites (DA, DOPAC, HVA, 5-HT or 5-HIAA) were determined only in L-DOPA-treated MPTP-lesioned NHP and human samples. Briefly, samples were sonicated in 0.4 M PCA, supernatants filtered and metabolites analyzed by HPLC in the filtrates.

#### Statistical analysis

Standard parametric t-test was applied to analyze AADC activity in rat, human and NHP samples. Mann-Whitney U-test was applied to analyze NHP data. Significance was defined as a p-value of less than 0.05.

## Results

### Clinical characteristics of Parkinson’s disease patients

Neuropathological analyses of all PD patients showed severe degeneration of substantia nigra, presence of Lewy bodies in remaining nigral neurons and gliosis. Ten PD patients were identified as responsive to L-DOPA, but the interval between their last L-DOPA (Sinemet) dose and death was not recorded. In 2 PD patients, responsiveness to L-DOPA was recorded as questionable. HPLC analysis indicated generally low levels of L-DOPA in analyzed brain regions both in PD patients and in control subjects.

However, 4 PD patients had high concentrations of L-DOPA in striatal subdivisions and oC compared with controls (data not shown). Because L-DOPA treatment may have down-regulatory effects on AADC [20, 21], these patients were excluded from further analysis. In the control group, 2 subjects evinced almost undetectable levels of striatal monoamines and were also eliminated from analysis, since these analytes were required for assuring *post-mortem* tissue integrity.

### Striatal AADC activity and DA metabolism

AADC activity measured in parkinsonian and control striatal tissue extracts from rats, NHP and humans is summarized in Table 2. For interspecies comparison of striatal AADC activity, we pooled the results obtained from different striatal regions for NHP and human putamen.

**Table 2:** Striatal AADC activity (pmol/mg protein/min) in rats, NHP and humans.

Species	Control		Parkinsonian		PC-Change^1^ (%)
	n	AADC Activity	n	AADC Activity	
Rat	7	277.48 ± 13.20	7	73.35 ± 4.04	- 73.5***
NHP	2	163.04 ± 7.95	2	12.84 ± 1.27	- 91.9**
Human^2^	8	7.01 ± 1.06	8	0.89 ± 0.09	- 87.3**

Data expressed in Mean ± Standard deviation.

^1^Difference between parkinsonian and control AADC activity values.

^2^Values came from sorted, raw pooled samples from different regions of the human putamen. Unpaired, two-tailed t-test

** p < 0.001.

*** p ≤ 0.0001—compared to corresponding control subjects.

Mean striatal AADC activity in rat, NHP and human differed greatly. The greatest AADC activity was observed in rats, followed by that in NHP, which was almost half that seen in rat (p<0.01), and then by human striatum, which was 40-fold lower than rat (p<0.0001). The difference between primates was also statistically significant since AADC activity in NHP was 20-fold higher than mean human levels (p<0.001). As expected, the development of parkinsonian neuropathology in striatum, either naturally or by administration of a neurotoxin, was associated with significant reduction of AADC activity, regardless of the species (Table 2). However, rats showed less severe depletion of AADC than either MPTP-lesioned NHP or PD subjects did relative to their respective controls. Severe lesioning of rat striatum by 6-OHDA led to a mean 73.5% decrease, whereas parkinsonian NHP and human putamen AADC content was reduced by 92% and 87%, respectively. Our findings clearly show dramatically lowered AADC activity in the degenerated striatum of PD patients, concomitant with data from neurotoxin-induced rodent and NHP models.

**Fig 1:**
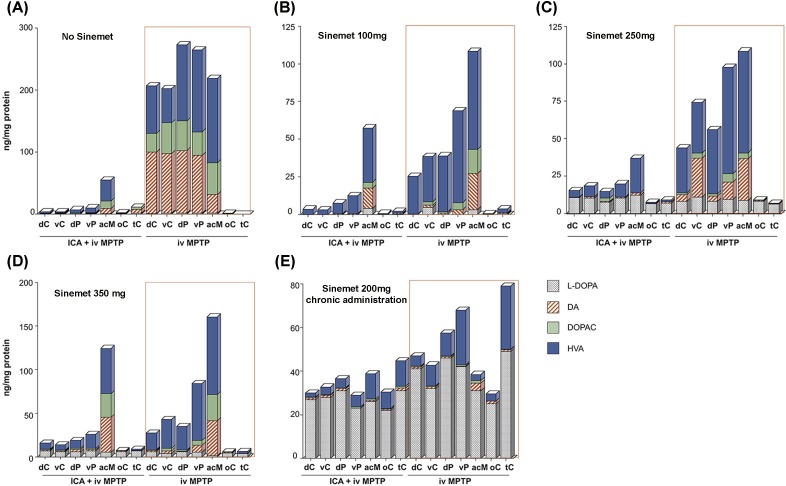
Dopamine metabolism after L-DOPA treatment in overlesioned MPTP monkeys. Dopamine and its metabolites were evaluated in different regions of the striatum of asymmetrically parkinsonian NHP that had been administered different doses of Sinemet 45 min prior to necropsy. Each graph shows data obtained from an individual animal receiving a different Sinemet dose. HPLC analysis of the different brain regions revealed that the severely lesioned side (ICA + iv MPTP) was almost completely devoid of DA and its metabolites compared to the partially lesioned side (iv MPTP), in which reduction was also milder in most of the regions analyzed (A). Analysis of the same regions in MPTP animals euthanized 45 min after the last dose of Sinemet indicated a similarly reduced conversion of L-DOPA into DA to that seen in the control animal (A), regardless of the dose of Sinemet received [(B) Sinemet 100 mg, (C) Sinemet 250 mg or (D) Sinemet 350 mg]. Another animal was chronically treated for 2 months with 200 mg Sinemet daily and euthanized 45 min after the last Sinemet dose (E). This animal had higher L-DOPA levels on both sides, whereas DA levels remained as low as those in the acutely challenged animals. Abbreviations: acM: nucleus accumbens; dC: dorsal caudate nucleus dP: dorsal putamen; ICA: intracarotid artery; iv: intravenous; vC: ventral caudate nucleus; vP: ventral putamen; oC: temporal cortex; tC: temporal cortex.

We previously showed [9, 22] that the severely lesioned striatum of NHP is unable to efficiently decarboxylate exogenous L-DOPA to DA and this was further established in the present study. We used HPLC to determine L-DOPA, DA and its metabolite content in different striatal regions of 4 asymmetrically MPTP-lesioned NHP (Fig 1A–1D). Tissue was isolated 45 min after acute L-DOPA treatment (Sinemet) and the analysis showed that, regardless of the L-DOPA dose administered (100, 250 or 350 mg of Sinemet), conversion of L-DOPA into DA was very inefficient on the severely lesioned side, whereas on the partially lesioned side (less affected by MPTP intoxication) some DA was detected, mostly in acM and dC. Furthermore, we studied L-DOPA to DA conversion in the NHP model mimicking more closely the PD striatum; that is, a striatum severely depleted of DA and chronically receiving L-DOPA medication. Data from a severely parkinsonian NHP placed on chronic Sinemet medication (200 mg daily) revealed that, in all structures tested 45 min after last administration, L-DOPA levels were very high but almost no DA was detectable, suggesting there was very limited L-DOPA decarboxylation in the lesioned brain areas analyzed (Fig 1E).

**Fig 2:**
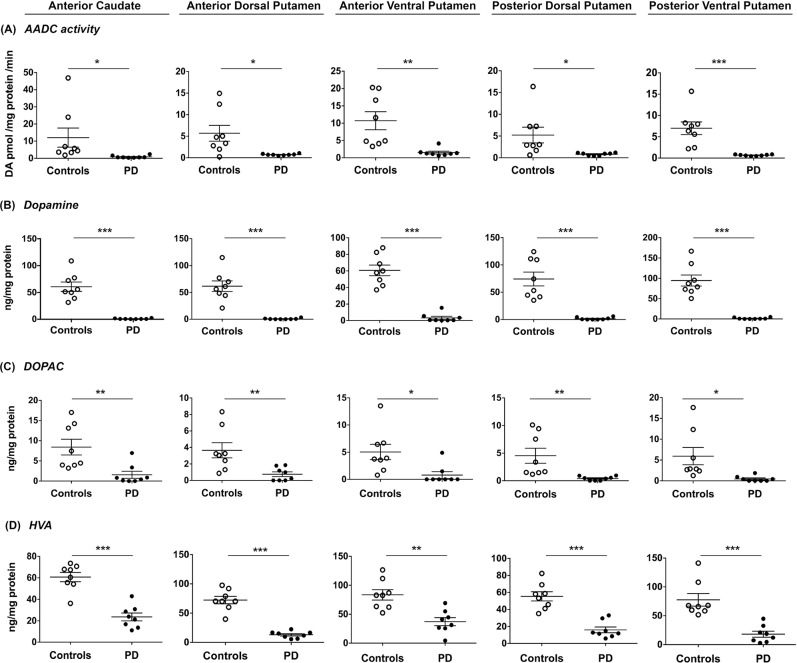
Dopaminergic metabolites in Parkinson’s patients and age-matched controls. AADC activity (A), dopamine levels (B), DOPAC levels (C) and HVA (D) levels in anterior portion of the caudate nucleus and different sub-regions of the putamen in control subjects (**o,** n = 8) and patients with PD (•, n = 8). Reduction of AADC activity was accompanied by greatly reduced levels of DA and its metabolites throughout the putamen. Caudate nucleus exhibited the same magnitude of reduction observed in putamen. Scatter plots depict mean ± SEM for each cohort and differences were analyzed between groups by unpaired, two-tailed t-test. *: p ≤ 0.05; **: p ≤ 0.01, ***: p ≤ 0.001.

To confirm PD-related striatal pathology in monoamine metabolism, we regionalized putaminal sample collection and examined anterior-posterior and ventral-dorsal distribution of AADC activity, DA, DOPAC and HVA in PD patients and control subjects. Regardless of the region of putamen analyzed, we detected significantly reduced AADC activity and levels of DA and its metabolites, DOPAC and HVA, in all PD patients compared to control subjects (S1 Table). Mean AADC activity was uniformly decreased (p<0.001) in all putaminal areas of PD patients compared to controls (adP, - 87.7%, avP, - 86.5%, pdP– 85.6%, pvP -90.1%). Accordingly, DA content was also severely reduced by >95% (p<0.001) in PD patients (S1 Table). Putaminal DOPAC levels in those patients were decreased by more than 80% compared to controls (p<0.001) and no significant regional differences were observed. Putaminal HVA in PD patients was also markedly depleted. In the putaminal regions analyzed, most PD patients had values below the lower limit of the control range for AADC activity, DOPAC and HVA in comparison to control subjects (Fig 2A, 2C and 2D), and there was no overlap in the ranges of DA content in PD patients and controls (Fig 2B).

**Fig 3:**
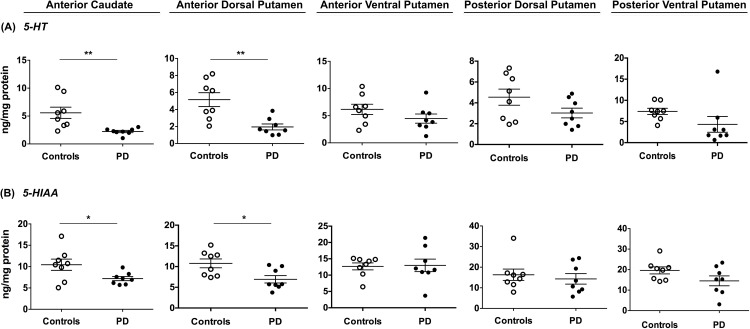
Serotonergic metabolism in Parkinson’s disease patients and age-matched controls. Levels of serotonin (A) and its metabolite, 5-HIAA (B) were measured by HPLC in the anterior caudate nucleus and different sub-regions of the putamen in control subjects (**o**, n = 8) and patients with PD (•, n = 8). All regions analyzed showed serotonin and 5-HIAA data overlap between control and PD groups and were only significantly reduced in the anterior dorsal putamen and the anterior caudate nucleus. Scattered plots depict mean ± SEM for each cohort and differences were analyzed between groups by unpaired, two-tailed t-test. *: p ≤ 0.05; **: p ≤ 0.01, ***: p ≤ 0.001.

Interestingly, a similar profile was found in the caudate nucleus of PD patients. AADC activity in the aC was significantly reduced, by 93% (p<0.001) (S1 Table), coupled with an equally significant reduction in DA (91.4%; p<0.001), DOPAC (81.4%; p<0.001) and HVA (61%; p<0.001). As shown in Fig 3, the reduction in AADC activity as well as in DA and its metabolites was very similar to that in putamen, indicating that the dopaminergic degeneration intrinsic to PD affects caudate nucleus to a very similar extent as it does putamen.

### Striatal serotonergic levels in PD

**Fig 4:**
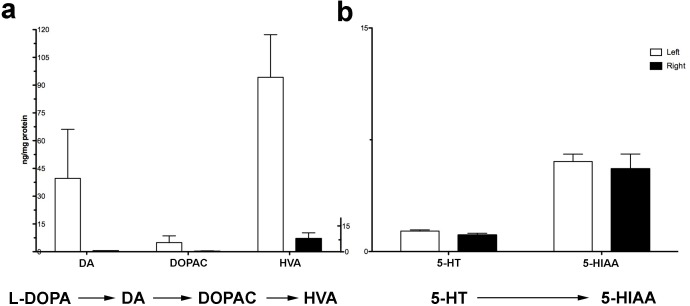
Monoamine levels measurement in overlesioned parkinsonian monkeys. MPTP administration resulted in an asymmetric parkinsonism in NHP presenting a more severe dopaminergic degeneration on the right striatum than that on the left. Accordingly, analysis of monoamine levels by HPLC revealed that there was a dramatic reduction of DA and its metabolites in the more severely lesioned putamen (right) compared to the mildly lesioned one (left) (a). In contrast, serotonin and its metabolite levels, measured in the same samples, were similarly depleted in both hemispheres (b).

Serotonin (5-HT) reduction has been observed in previous PD studies, and it has been suggested to play a role in L-DOPA-induced dyskinesias [23] and the loss of response to L-DOPA [24]. In the present study, we measured 5-HT and 5-HIAA levels in our human samples and found a reduction similar to that previously described. This reduction of striatal 5-HT and 5-HIAA was on average less severe than that observed in DA and its metabolites in PD patients compared to matched controls (S1 Table). Serotonin and 5-HIAA levels in putamen showed greater overlap in individual ranges and there was no significant reduction in any region but the adP and caudate nucleus, which exhibited 5-HT reduced by ~60% (p<0.01) and 5-HIAA by ~33% (p<0.01) in PD (Fig 3). In a previous study, we chronically administered L-DOPA to MPTP-lesioned animals that received either adeno-associated virus encoding human AADC gene (AAV2-hAADC) or PBS [13]. We measured 5-HT and its metabolite in the PBS group because, since it remained untreated, it mimicked the striatal condition of PD patients who are taking L-DOPA. The effect of MPTP on monoamine levels in our NHP were examined. Neither 5-HT nor its major metabolite, measured in the same samples that were analyzed for DA and its metabolites (Fig 4)**,** were significantly reduced in the severely MPTP-lesioned NHP striatum compared to the partially lesioned contralateral side (p>0.50, Fig 4). Thus, lack of efficient conversion of L-DOPA to DA in MPTP-lesioned NHP cannot be explained by loss of serotonergic innervation and strongly indicates that AADC in nigrostriatal fibers (which are dramatically reduced by MPTP in NHP and in PD patients) play the predominant role in L-DOPA to DA conversion.

## Discussion

PD patients suffer a progressive depletion of dopaminergic function and synthetic enzymes, such as tyrosine hydroxylase and AADC, in basal ganglia and in other catecholaminergic tissues. However, the loss of dopaminergic function appears to be uneven and previous studies have suggested that the caudate nucleus is relatively spared in PD [13]. However, this conclusion is based more on PET and SPECT imaging in living subjects than on direct measurement [25]. In this study, we assayed AADC activity and monoamine levels in striatum of PD patients. We detected considerable depletion (>80%) of AADC and monoamine levels throughout striatum in PD patients. In contrast, as measured in the same regions as DA and its metabolites, 5-HT metabolism were much less affected by PD in accordance with the findings by others [14]. It is interesting that, even though there was a >80% reduction in AADC activity in PD brains, tryptophan was still converted to 5-HT in the same striatal regions in which DA levels were dramatically reduced. Based on this fact, lack of efficient conversion of L-DOPA to DA in MPTP-lesioned NHP was clearly not due to any dramatic decrease in 5-HT, but by the loss of AADC in the nigrostriatal fibers damaged by MPTP in NHP.

Mean disease duration in PD subjects was 13.1 ± 4.8 years after diagnosis, but almost all subjects were recorded as L-DOPA-responsive. This latter parameter is especially important since it indicates that, although advanced, these were not end-stage patients. A major problem in analyzing *post-mortem* brain tissues is detection of potential confounds. We excluded 4 PD subjects because, in contrast to the negligible level in the rest of the cohort, their striatal L-DOPA content was unusually high and it was unclear how this variable would affect our biochemical assays.

Anterior-posterior and dorsal-ventral *post-mortem* analysis of PD patients revealed severe (≥85.6%) depletion of AADC activity in putamen, regardless of the putaminal sub-region, in line with a dramatic loss of DA (>95.8%). Our results agree with previous studies of putaminal AADC activity in advanced PD [26, 27]. However, these studies did not examine in detail sub-regional putaminal AADC activity. PET and SPECT imaging in the parkinsonian brain, as well as *post-mortem* investigations, demonstrated a more pronounced loss of dopaminergic markers in posterior putamen [13, 28]. This phenomenon can be explained by a predominant degeneration of the ventral tier of the substantia nigra that projects to the posterior part of the putamen [29, 30]. However, severe depletion was also observed in the rostral sector of this nucleus [31]. Our detailed biochemical analysis revealed that, in advanced PD, dopaminergic markers are slightly more depleted in adP than in the avP (-98.9% *vs*. -95.8% of DA levels compared to controls, respectively). The number of human sample is too small to allow us draw any definite conclusions regarding the intra-regional difference in dopaminergic loss. Nevertheless, regardless of the putative intra-regional difference, the >95% loss of DA far exceeds the “clinically relevant threshold”, ~80% of striatal dopamine depletion, that results in functional decompensation and manifestation of PD.

Unexpectedly, we also detected robust reduction (>90%) in all dopaminergic markers (AADC activity, DA and metabolites) in aC. It has been assumed that there is a fairly specific pattern of loss of striatal dopaminergic markers in PD, with less severe dopaminergic pathology in caudate versus putamen [32]. However, the relative sparing of caudate nucleus in PD may be limited to specific regions of this nucleus. This suggestion is supported by studies [14, 33] showing that DA reduction in PD is more marked in dorsal-rostral caudate nucleus than in the ventral caudate nucleus. Our results indicate that the anterior segment of caudate nucleus (head) is the only part of this structure with DA loss exceeding 80%.

Although the anterior-posterior gradient of DA loss in caudate nucleus is not fully understood, it has specific clinical consequences. The striatum represents the primary input station of the cortico-striatal-thalamo-cortical circuits [34] and is functionally organized into limbic, associative and sensorimotor subdivisions. The post-commissural putamen is involved in motor functions via connection with sensorimotor cortices. The caudate nucleus and pre-commissural putamen, through their connections with associative cortices, participate in processing of information related to cognitive function [34, 35]. The heterogeneous structure of the striatum [36] suggests regional effects of DA dysfunction on the manifestation of the cardinal motor and non-motor symptoms of PD. SPECT imaging of DA transporter (DAT) showed that DAT loss in striatum of PD patients is associated with clinically important long-term motor and non-motor signs of PD [21]. PD patients with lower DAT were more likely develop severe behavioral and cognitive disabilities [21]. Another study used SPECT DAT imaging to demonstrate that depressive symptoms of PD were associated with lower DAT in caudate, whereas motor symptoms were associated with decreased DAT in putamen [31]. These results suggest that both motor and non-motor symptoms of PD are associated with severe dopaminergic abnormalities in different subdivisions of striatum. This view is further supported by the fact that depression in non-treated PD patients often recedes after initiation of the L-DOPA treatment [37, 38].

Our group has been working for a long time on increasing AADC levels in the striatum of PD patients by means of gene therapy with an AAV2 carrying hAADC gene. Transduction of striatal cells with AAV2-hAADC aims to restore the brain’s ability to convert L-DOPA to DA, which in PD patients would result in a larger fraction of L-DOPA medication being converted to DA. In the context of AAV2-hAADC gene therapy, previous and current clinical studies have been focused on targeting of the AADC transgene to the posterior (post-commissural) putamen to ameliorate the motor deficits of PD. However, progressive degeneration of other subdivisions of striatum related to non-motor dysfunctions in PD [39, 40] suggests that a broader strategy makes sense in the light of the present data.

In the present investigation, the AADC activity data from PD patients was compared with rodent and NHP neurotoxicant models of PD. Although a number of reports have investigated the influence of PD on striatal AADC activity [26, 41], to our knowledge, no prior study has directly compared data from three different species and brain regions. We showed that AADC in healthy striatum varies widely across species [41] with the highest levels of AADC in rat striatum and the least in human tissue. The reason for this species difference is unclear, but may be partially explained by different motor behavior strategies. Experimental rodent and NHP models of PD mirror the DA deficiency in advanced PD [7, 16, 19]. In both human PD and lesioned NHP the magnitude of AADC activity loss was similarly severe and roughly paralleled the changes in DA. In conclusion, this study supports the concept that expanding AADC gene transfer into caudate nucleus in addition to the presently targeted putamen [10, 12] and may result in further increase of clinical benefits, such as cognitive deficit.

## Supporting information

S1 Table AADC activity (pmol/mg protein/min) and monoamine (ng/mg protein) levels of control and PD human brain tissue. (PDF)

## Abbreviations

PD: Parkinson’s disease
AADC: Aromatic L-amino acid decarboxylase
DA: Dopamine
DOPAC: 3,4-Dihydroxyphenylacetic acid
HVA: homovanillic acid
5-HT: 5-hydroxytryptamine
5-HIAA: 5-Hydroxyindoleacetic acid
NHP: Nonhuman primate
