# Supplementary material for: Depletion of AADC activity in caudate nucleus and putamen of Parkinson’s disease patients; implications for ongoing AAV2-AADC gene therapy trial
Source: PLoS One. 2017 Feb 6;12(2):e0169965. doi: 10.1371/journal.pone.0169965 (PMC5293261; doi:10.1371/journal.pone.0169965)
Supplement: S1 Table — (PDF) [file pone.0169965.s001.pdf]

Supplemental Table 1: AADC activity (pmol/mg protein/min) and monoamine (ng/mg protein) levels of control and PD human brain tissue.

	AADC	% red.	DA	% red.	DOPAC	% red.	HVA	% red.	DA:HVA	5-HT	% red.	5-HIAA	% red.
Anterior caudate													
PD	0.85±0.26*	-93.0	5.45±2.74***	-91.0	1.57±0.86**	-81.4	23.63±3.68***	-61.6	0.23	2.23±0.21**	-60.0	7.19±0.48*	-31.1
Controls	12.06±5.56		60.83±8.71		8.42±1.95		60.74±4.28		1	5.58±1.01		10.44±1.33	
Anterior dorsal putamen													
PD	0.72±0.06*	-87.3	0.71±0.41***	-98.8	0.75± .65**	-79.5	13.08±2.01***	-81.9	0.05	1.95±0.34**	-62.2	6.93±0.89*	-35.7
Controls	5.69±1.84		61.67±9.71		3.65±0.91		72.39±6.39		0.85	5.16±0.82		10.77±1.06	
Anterior ventral putamen													
PD	1.51±0.36**	-85.9	3.21±1.77***	-94.7	0.79±0.57*	-84.3	37.34±6.61***	-55.3	0.09	4.46±0.79	-27.6	12.98±1.81	2.4
Controls	10.69±2.46		60.64±6.04		5.04±1.32		83.55±8.41		0.73	6.16±0.89		12.67±0.99	
Posterior dorsal putamen													
PD	0.82±0.11*	-84.3	1.66±0.81***	-97.8	0.41±0.14**	-90.9	15.98±3.51***	-71.2	0.1	3.02±0.46	-33.5	14.33±2.56	-12.2
Controls	5.23±1.81		74.29±12.7		4.53±1.36		55.43±5.41		1.34	4.54±0.77		16.32±2.75	
Posterior ventral putamen													
PD	0.67±0.06***	-90.4	1.43±0.71***	-98.5	0.45±0.23*	-92.4	18.01±5.16***	-76.8	0.08	4.33±1.86	-41.4	14.52±2.41	-26.0
Controls	7.01±1.49		94.62±13.62		5.93±2.07		77.59±10.9		1.22	7.39±0.73		19.62±1.72	

Data are expressed as mean ± SEM.
% red.: percentage of reduction of the analyte in the PD patients compared with the healthy controls
Unpaired, two-tailed t-test: * $p \leq 0.05$; ** $p \leq 0.01$; *** $p \leq 0.001$ – compared to healthy controls
